# Modern Sociology

**Published:** 1901-11-23

**Authors:** 


### Modern Sociology.

### THE PREVENTION OF HYDROPHOBIA.

Rabies in dogs, and by consequence hydrophobia in human beings, has been driven out of England and Scotland. The steady enforcement of the muzzling order at the first news of rabies has prevented animals so affected biting other animals or men, and a horrible disease, which until lately claimed always many humbler and a few human victims, is checked. There was, however, a mysterious outbreak of rabies in Wales last year, which caused the death of several animals, though fortunately no human being was attacked. Early in the year, the officials of the Veterinary Department of the Board of Agriculture received reports of suspicious cases, but the evidence was not sufficient to justify the issuing of a muzzling order. But on July 9th an unknown stray dog died with suspicious symptoms, after having bitten several other animals. As a precaution, the animals thus bitten were isolated and kept under observation. One of these showed such marked symptoms of rabies, that on August 21st the owner shot it, and reported his opinion to the police. Post-mortem examination showing clearly that this was a true case of rabies, a muzzling order was at once issued, covering great part of the counties of Carmarthen, Glamorgan, and Brecon. This checked the disease, though not immediately, for a stray sheep-dog, which it was nobody's personal business to muzzle, managed to quarrel with and bite a dog at a farm it visited, and this in turn bit several cattle and a mare before it was destroyed, all of which animals died of rabies. However, the epidemic was checked before any great mischief was done, and, above all, before any human being had fallen a victim.

But whence came the first infection? That has not been found out, but it is surmised that it was brought from the Continent, where rabies is yet common, by some dog, perhaps the pet of a sea captain, which came to one of the Welsh seaports. It is difficult, especially amidst the bustle of mooring and unloading a vessel, to watch a dog. Perhaps the animal ought to be tied up in some part of the ship, but it very seldom is. Very naturally it wants to stretch its legs by a run on shore, and not unnaturally, especially if it is rendered irritable by a poison in its blood, it quarrels with and bites some dog it meets, and so the disease is established. In view of this danger, therefore, it has been found necessary to regulate carefully the importation of dogs into this country from abroad. It may not be altogether possible to prevent such an accident as we have supposed may have caused this Welsh outbreak, but it is possible to prevent infected dogs from abroad taking up a permanent residence in this country and spreading infection before their condition is suspected. An order, therefore, is enforced that all dogs coming from abroad should undergo a period of six months' quarantine. To meet the requirements of this order, extensive kennels at Beddington Lane, near Mitcham, have been set apart as an isolation station for foreign dogs.

As might be guessed, this order meets with great disapprobation from the owners of pet dogs. Having a personal affection for their animals, they feel it a grievance to be parted from them for six months, before the end of which time Dash and Fido may have forgotten the hand that once fed them, or at least will have missed their former social advantages and lapsed into the ways of common dogs. In such cases a modification of the order is allowed. No foreign dog may be landed in this country without a licence for it being obtained, but dogs which have been under the personal care of their owners for the three months immediately preceding the date of application for this licence are allowed to land, but must then be isolated in a house of which the owner is the householder. Flats and lodgings are not regarded as suitable places for the isolation of dogs. It is also ordained that no other dog shall be kept on the premises while the dog from abroad is under quarantine. The objection to the detention of dogs in houses of which the owner of the dog is not the householder, arises from the inability of the applicant for a licence to give a satisfactory guarantee that the dog will be detained in one place for the full period of quarantine; and in view of the difficulty of maintaining the necessary supervision unless the dog completes its period of isolation in one place, it has been found necessary to limit the issue of a licence to cases in which the owner of the dog can undertake that this will be done. If the applicant has no fixed place of residence in Great Britain, arrangements must be made for the detention of the dog on the premises of an experienced veterinary surgeon. Licence to remove a dog before the period of its isolation is over is given only under exceptional circumstances, and, unless arrangements for its completing its quarantine can be shown to be as satisfactory as those in the place where the isolation first began, it must be sent to the care of a veterinary surgeon. It is never permitted to remove a dog from a rural district to a town. The Department of Agriculture are not sure that even these regulations are strict enough, and that it would not be better to insist on the six months' isolation of all dogs brought from abroad on the premises of some experienced veterinary surgeon. On one thing at least the board insists—namely, that dogs shall not be brought from abroad by persons coming on a short visit to this country. "They do not think they would be justified in exposing dog owners in this country to the risk of re-introducing the disease, however small it may be in any individual case, merely for the convenience of persons visiting this country for short periods." They also recommend that the owners of pet dogs should wrench themselves from their favourites when they pay a visit to the Continent, and thus avoid the risk of the dogs being infected there, in addition to all the trouble of obtaining a licence and isolating the animal when they come home.

Some modification of the rule is permitted in the case of trick or performing dogs. It is impossible that these animals, who earn their masters' living, can be isolated for six months, and in the case of such dogs the presumption is that, for that very reason, the masters will be careful to protect them from infection. But it is at least insisted on that these troupes shall not come into contact with other dogs, and that they shall be kept muzzled except when actually engaged in their performance. Another point of general interest is the admission of high-class dogs from the Continent to take part in dog shows. In response to representations which were received from the Kennel Club and the International Kennel Club, arrangements have been made which permit the exhibition of such dogs in cases where the authorities of the show are prepared to adopt very stringent precautions to prevent the possibility of the imported dogs coming into contact with British dogs, either in the showyard or ring. This was done at the Alexandra Palace Show in September, 1900, and no harm has resulted from the concession. Satisfactory as it is to find that the efforts of the Board of Agriculture have been so successful in stamping out hydrophobia and bringing rabies under control, the stringency of the measures which have been found necessary for the purpose, including as they have done muzzling over large areas and the wholesale destruction of suspected animals, shows very conclusively how little reason we have for believing that any such measures of isolation as could be enforced on human beings could stamp out any of the infectious diseases to which mankind is subject.

